# Feeding Management and Albendazole Pharmacokinetics in Pigs

**DOI:** 10.3390/ani13030474

**Published:** 2023-01-30

**Authors:** Alvarez Luis Ignacio, Chiappetta Valentina, Moriones Lucila, Dominguez Paula, Cantón Candela, Lanusse Carlos, Ceballos Laura

**Affiliations:** Laboratorio de Farmacología, Centro de Investigación Veterinaria de Tandil (CIVETAN), UNCPBA-CICPBA-CONICET, Campus Universitario, Tandil CP 7000, Argentina

**Keywords:** albendazole, pharmacokinetics, systemic exposure

## Abstract

**Simple Summary:**

Helminth parasite infections have a significant impact on the efficiency of swine production as a consequence of the reduced feed conversion and weight gain and the condemnation of affected organs after slaughter. Parasite control is primarily based on the use of chemical agents, such as albendazole, a broad-spectrum benzimidazole anthelmintic compound. After oral administration, the dissolution and absorption of albendazole may be significantly modified by the type of diet, which may induce substantial variation in the amounts of the active drug/metabolites that actually reach the parasite to exert the anthelmintic action. This diet-related variability may have relevant implications on the drug efficacy, affecting the overall success of parasite control programs. Herein, we describe the relationship between the feeding management and the pharmacokinetic behavior of albendazole in pigs. Although the type of diet did not result in a significant difference in the systemic exposure to albendazole/metabolites, the higher fat content in the diet (by the addition of soya oil) was correlated with higher albendazole sulfoxide concentrations 12 h post-treatment.

**Abstract:**

Albendazole (ABZ) is a methylcarbamate benzimidazole anthelmintic used to control gastrointestinal parasites in several animal species and humans. The type of diet has been identified as a major determinant for ABZ pharmacokinetics in different animal species and humans. The work described here assesses the pattern of the absorption and the systemic availability of ABZ and its metabolites after its oral administration to pigs under different feed management plans. Eighteen pigs (5 months old, local ecotype breeds) were distributed into three experimental groups. In the fasting group, the animals fasted for 8 h prior to treatment. In the pellet + oil and pellet groups, the animals were fed ad libitum with a commercial pelleted-based diet with or without the addition of soya oil. An ABZ suspension was orally administered at 10 mg/kg. Blood samples were taken over the 48 h post-treatment. The plasma samples were analyzed by HPLC. Under the described experimental conditions, the ingestion of the pellet-based diet with or without the soya oil before ABZ treatment did not significantly (*p* < 0.05) modify the plasma disposition kinetics of the ABZ sulfoxide (ABZSO, the main ABZ metabolite) compared to that observed in the fasting pigs. Both ABZ metabolites (ABZSO and ABZ sulphone) reached similar peak concentrations and systemic exposures in all the experimental groups regardless of the feeding management. However, the addition of oil to the pelleted food enhanced the pattern of ABZ absorption, which was reflected in the higher (*p* < 0.05) concentration profiles of the active ABZSO metabolite measured between 12 and 48 h post-treatment compared to the pigs fed with the pelleted food alone. Although this effect may not be therapeutically relevant after ABZ administration as a single oral dose, the overall impact of the type and feeding conditions when ABZ is supplemented with food for several days should be cautiously evaluated.

## 1. Introduction

The impact of parasitism on the efficiency of pig production continues to be a worldwide concern [1]. The nematodes *Ascaris suum*, *Trichuris suis,* and *Oesophagostomum* spp. are among the most common helminth species affecting swine production [2]. These infections may lead to reduced weight gain, poor feed conversion, and even organ damage by migrating larvae, which in turn may result in condemnations at slaughter [3].

The control of gastrointestinal (GI) nematode infections in livestock is primarily based on the use of chemotherapy, with the risk of leading to the development of nematode resistance due to inappropriate drug use [4].

Drugs belonging to the benzimidazole (BZD) and macrocyclic lactone groups are among the most widely used for the control of nematodes in pigs [5]. The BZD compounds were introduced into the medicine veterinary market primarily for the control of helminth parasites in domestic animals [6]. Their widespread use, particularly the methylcarbamate derivatives such as albendazole (ABZ), is based on their high efficacy, low toxicity, and broad spectrum of activity [7]. BZD activity depends on the capacity to reach high and time-sustained concentrations at the site of the parasite [8]. The optimum activity is achieved when the target parasite is exposed to the effective drug for an extended period of time. Active drug/metabolites will be available to diffuse through the cuticle of the nematode, bind to its specific receptor (ß-tubulin), and cause the therapeutic effect [9].

As a methylcarbamate compound, ABZ has poor water solubility. The acidic pH of the stomach facilitates the ABZ dissolution and subsequent absorption in the lower gastrointestinal tract [10,11]. The time that the ABZ remains in the stomach may affect the amount of the solubilized drug’s absorption and resultant pharmacokinetic behavior [10]. Any factor that consistently influences the gastric emptying rate may also have a profound effect on the rate and extent of drug absorption in monogastric species [12,13]. As previously reported [12], the type of feeding affected the systemic availability of ABZ metabolites in pigs. In that context, the fed/fasting condition has been postulated as a strategy to improve the ABZ dissolution and optimize the systemic exposure and efficacy in different ruminant species [12,14,15,16]. In addition, the type of diet may affect the flow rate of the digesta, affecting the pharmacokinetics of the orally delivered drugs [17]. This is a factor to consider in pig production systems, where it is common to use different feeding and management conditions.

The pharmacokinetic behavior of ABZ has been studied in several species including sheep [18,19], cattle [8,20], goats [21,22], pigs [12], donkeys [23], dogs [24], and humans [25,26]. The ABZ sulfoxide (ABZSO) active metabolite and the sulphone (ABZSO_2_) inactive derivative are the main products found systemically after ABZ administration. The parent drug is extensively metabolized mainly in the liver [27] but also in the extrahepatic tissues [28,29].

In order to optimize the therapeutic response, since the development of resistance to drugs is becoming a severe threat to the control of parasites, the objective of the current work was to evaluate the pattern of the absorption and the systemic availability of ABZ and its metabolites after oral administration to pigs under different feeding management plans (fasting before treatment or fed a pellet-based diet with or without the addition of soybean oil).

## 2. Materials and Methods

### 2.1. Chemicals

ABZ, ABZSO, and ABZSO_2_ (99% purity) were purchased from Toronto Chemicals Research Inc. (Toronto, ON, Canada). Oxibendazole (OBZ), used as the internal standard, was from Sigma-Aldrich (St. Louis, MO, USA). The HPLC grade solvents, acetonitrile and methanol were purchased from Baker, Mallinckrodt (Baker, Phillipsburg, NJ, USA). The ABZ formulation administered to the pigs was Overzol^®^ 10% (Laboratorios Over SRL, Argentina).

### 2.2. Animals

The study was conducted in eighteen crossbred pigs (26.3 ± 4.7 kg, 5 months old, obtained from a local pig farm). The animals were fed ad libitum with a commercial pelleted-balanced feed (Cerdo iniciador^®^, Cooperativa Agropecuaria, Tandil, Argentina), composed of 17% crude protein, 3% ethereal extract, 6% crude fiber, 10% maximum humidity, and an energetic value of 3300 K·cal/kg of dry matter. A 10 day acclimatization period was allowed for adaptation. The animals were housed at room temperature in pens with concrete floors protected from rain with free access to water. Animal procedures and management protocols were carried out in accordance with the Animal Welfare Policy (Act 087/02, protocol number 08/21) of the Faculty of Veterinary Medicine, Universidad Nacional del Centro de la Provincia de Buenos Aires (UNCPBA), Tandil, Argentina and internationally accepted animal welfare guidelines [30].

### 2.3. Experimental Design, Treatments, and Sampling

The experimental animals were randomly assigned to 1 of 3 experimental groups and housed in different pens. In the fasting group, the animals were subjected to an 8 h fasting period prior to ABZ administration, (water was provided ad libitum even during the fasting period); in the pellet + oil group, the animals were fed ad libitum with a commercial pelleted-balanced complete food mixed with soya oil (soya oil final concentration 8%); and in the pellet group, animals were fed ad libitum with a commercial pelleted-balanced complete food. All animals belonging to the different experimental groups were orally treated with the ABZ suspension formulation at the dose of 10 mg/kg. Blood samples were taken before treatment (time = 0) and 2, 4, 6, 8, 10, 12, 24, 30, 36, and 48 h post administration, following the procedure reported by Alvarez et al. [12]. The blood samples were centrifuged at 2500× *g* for 15 min, and the plasma obtained was stored at −20 °C until it was analyzed by high-performance liquid chromatography (HPLC) [12].

### 2.4. Analytical Procedures

ABZ, ABZSO, and ABZSO_2_ were extracted from the samples (0.25 mL, spiked with 10 μL of OBZ 40 μg/mL) by the addition of 1 mL of acetonitrile. After a high-speed vortexing shaker (VWR Scientific Products, West Chester, PA, USA) over 15 min and centrifugation (2500× *g*, 15 min, 4 °C), the clear supernatant was evaporated to dryness under a gentle stream of nitrogen at 56 °C in a water bath (Zymark TurboVap LV evaporator, American Laboratory Trading, Inc., Lyme, CT, USA). The dry residue was dissolved in 150 μL of mobile phase and shaken (10 min) prior to injection in the HPLC system.

The experimental and fortified plasma samples were analyzed for ABZ, ABZSO, and ABZSO_2_ by HPLC. The equipment characteristics and chromatographic method were the same as those described in Ceballos et al. [19]

A complete validation of the analytical methodologies was performed following internationally recognized criteria [31] before starting the measurement of the analytes’ concentrations in the plasma samples from pigs. The compounds were identified using the retention times of pure reference standards. The calibration curves for the ABZ, ABZSO, and ABZSO_2_ in the plasma from pigs were prepared by least squares linear regression analysis, with ranges between 0.02 and 3 µg/mL with correlation coefficients > 0.996. The mean absolute percentage recoveries for ABZ and its metabolites were ≥89%. The limit of detection (LOD) was 0.008, 0.001, and 0.05 for the ABZSO, ABZSO_2_, and ABZ, respectively. The limit of quantification (LOQ) was 0.02 µg/mL. The concentration values below the LOQ were not considered for the pharmacokinetic analysis.

The pharmacokinetic analysis and the calculation of the parameters were carried out as described in Ceballos et al. [19].

The data are expressed as the arithmetic mean ± standard deviation (SD). Parametric tests (analysis of variance [ANOVA] plus Tukey) were used for the statistical comparison of the pharmacokinetic parameters obtained for the different experimental groups. A value of *p* < 0.05 was considered statistically significant, using Graph Pad Software, San Diego, CA, USA).

## 3. Results

**Figure 1:**
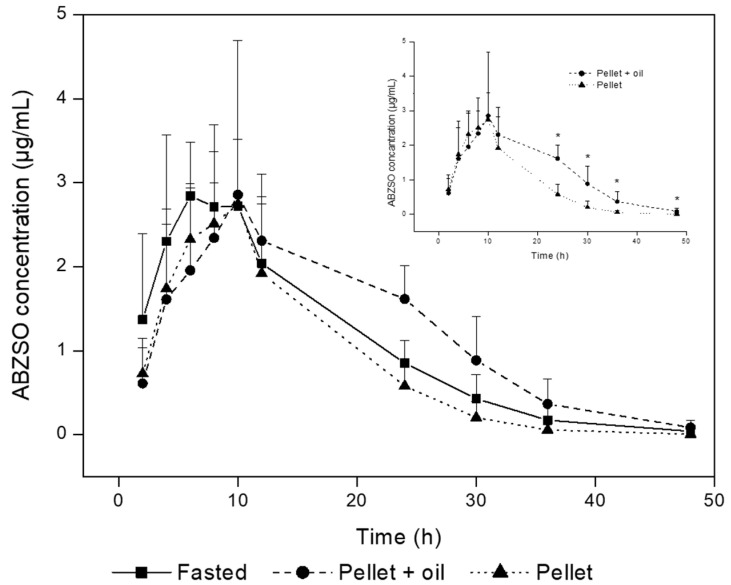
Comparative (mean ± SD) plasma concentration profiles of albendazole sulfoxide (ABZSO) obtained after albendazole suspension administration (10 mg/kg) to pigs either after 8 h fasting (fasting) or fed with a pellet + oil (pellet + oil) or pellet (pellet). The insert shows the comparative (mean ± SD) plasma concentration profiles for the ABZSO between the feeding groups. * Significant differences (*p* > 0.05).

None of the animals involved in the study showed any adverse events. The ABZ parent drug was not detected in the plasma of the treated animals. ABZSO was the main metabolite detected between 2 and 48 h post-treatment, follow by a lower concentration of ABZSO_2_. The comparative concentration profiles (mean ± SD) for ABZSO after the oral administration of ABZ (10 mg/kg) in pigs are shown in Figure 1. The ABZSO reached a similar Cmax (3 µg/mL approx.) at 7.0 ± 2.1; 12.0 ± 6.0, and 9.0 ± 2.1 h post-treatment for the fasting, pellet + oil, and pellet groups, respectively. Under the described experimental conditions, food ingestion before the ABZ treatment did not significantly (*p* < 0.05) affect the plasma disposition kinetics of the ABZSO in pigs. However, the addition of the oil in the food (pellet + oil group) was reflected in higher (*p* < 0.05) ABZSO concentrations from 24 to 48 h compared to the group fed with pellets alone (pellet group), which were obtained at a delayed Tmax. This result was reflected in a higher AUC_12–48h_ in the pellet + oil group (37.7 ± 8.5 µg·h/mL) compared to that in the pellet group (17.5 ± 9.1 µg·h/mL) and even in the fasting group (24.3 ± 7.2 µg·h/mL). Table 1 shows the main pharmacokinetic parameters obtained for the ABZSO after the oral administration of the ABZ in the different experimental groups. No statistical differences (*p* < 0.05) in the overall disposition kinetic were obtained between the pigs subjected to different diets/fasting prior to ABZ oral administration. The sampling schedule chosen provided a reliable estimation of the extent of the exposure since the AUC_0-LOQ_ covered ≥80% of AUC_0–∞_ in all the experimental groups (99% for the fasting and pellet groups and 97% for the pellet + oil group).

In both the fasting or pellet + oil groups, the ABZSO_2_ could be detected over the LOQ between 2 and 48 h, unlike the pellet group in which this metabolite was detected for a shorter period (4–36 h). However, no statistically significant differences (*p* > 0.05) were observed in the main pharmacokinetic parameters (Table 2) among the groups. The variation coefficients percentage (%CV) for the PK parameters was similar between all the experimental groups for the ABZSO and the ABZSO_2_ metabolites (24 to 37%).

## 4. Discussion

Although the ABZ parent drug, once dissolved, may be available to reach its main therapeutic targets (the nematodes located in the GI tract), only traces were detectable in the plasma of treated pigs, confirming the extensive liver first-pass oxidation [22]. As has been shown for many animal species including pigs [15], ABZSO and ABZSO_2_ were the main metabolites recovered in the bloodstream after the oral administration of ABZ as a suspension in all the experimental groups. Regardless of the feeding management, ABZSO was the main metabolite recovered in the pig plasma for a 48 h time period (Figure 1), which accounted for more than 75% of the total analytes recovered in the bloodstream in the treated animals.

As a chemical class, the BZD methylcarbamates have low water solubility, which is a serious limitation for their absorption. The drug that does not dissolve in the gastrointestinal contents passes down and is excreted in the feces without exerting its action. The water solubility of the BZD compounds drastically increases at extreme pH values [7]. Thus, the residence time of the ABZ suspension in the acidic stomach pH plays an essential role in its dissolution. The higher the length of time the drug remains in the stomach, the greater the amount that dissolves. Improved ABZ dissolution impacts its systemic and gastrointestinal exposure. This has been demonstrated in ruminants [12,25,32] and monogastric [15,33,34] species. In ruminants, the rumen acts as a drug reservoir, delaying the BZD outflow through the gastrointestinal tract. Previous studies have indicated that a short-term reduction in feed [33] or a fasting period [12,26] enhanced ABZ dissolution and absorption by delaying its permanence in the abomasum. In fact, the availabilities of ABZ and ABZSO in plasma and the gastrointestinal fluids/mucosae of fasting calves were markedly greater than in those fed ad libitum [12]. In monogastric species such as pigs, a shorter transit gastric time in animals fed grain-based diets was associated with lower ABZSO systemic exposure compared to animals fed high-fiber feed. High-fiber diets produce an increment in the gastric emptying time favoring ABZ dissolution in the acidic environment of the stomach [15].

Unlike the previous results obtained in ruminant species, fasting prior to treatment did not affect the systemic disposition of the ABZ metabolites in the pigs. In dogs, lower fenbendazole plasma concentrations were achieved when fenbendazole was given on an empty stomach, compared with its administration given in food [33], which was related to the differences in the gastric emptying time. However, in the current work, similar ABZSO plasma pharmacokinetic parameters (AUC, Cmax, or Tmax) were obtained in the pigs that fasted prior to treatment compared to those that were fed (pellet + oil and pellet groups). This was a puzzling result given that fasting should have increased the rate of gastric emptying. In fed animals, solids take a much longer time to be emptied from the stomach compared to fasting animals, impacting the ABZ dissolution and kinetics. Additionally, in the fasting state, the gastric juice consists of a solution of sodium chloride (NaCl) with a small amount of hydrochloric acid (HCl) and potassium chloride (KCl) [35]. In fed animals, associated with an increase in volume, the concentration of the HCl in gastric juice increases [35]. Under this condition, we would expect a higher ABZ dissolution in fed compared to fasting animals. There was not a clear explanation for the lack of the “fasting” effect observed in the pigs in the current work. One potential explanation could be related to the “Pavlov effect”. The cephalic influence on gastric acid secretion has been recognized since the beginning of the 20th century by the work of the Russian physiologist Ivan Pavlov, who conducted experiments with dogs. Pavlov’s work showed that the thought, sight, smell, and taste of food can evoke vagal excitation and lead to a gastric acid secretory response. Since the pigs in the fasting group were hosted close to both fed groups (fed 30 min before the ABZ treatment), it can be hypothesized that an increased acid secretion in fasting animals, as a consequence of the “Pavlov effect”, may have minimized the potential effect of fasting on ABZ dissolution. This hypothesis needs to be experimentally confirmed. However, fasting should have increased the gastric emptying rate affecting the ABZ dissolution, which was not observed. It is likely that another cause not considered here may have contributed to the observed dissolution/absorption/pharmacokinetic results.

Additionally, a modest effect on the systemic exposure to the ABZ metabolites was observed in pigs fed with a soya oil supplemented food (pellet + soya oil group) compared to that observed in animals fed only with pellets (pellet group). The addition of soya oil correlated with higher ABZSO concentrations after 12 h of treatment, with partial AUC12–48h values 44% higher than that observed in animals fed with pellets alone. Although similar values of Cmax, Tmax, and AUC_0-LOQ_ were observed in both groups, the differences observed in the partial AUC_12–48h_ were significant (*p* < 0.05). This statistically significant difference may not have any clinical consequence (especially considering the high interindividual variability) but would indicate the effect of food on the systemic disposition of ABZ. A fatty meal prior to treatment was associated with increased ABZSO systemic availability in ABZ-treated human volunteers [21]. The fatty composition of the diet was identified as a key factor enhancing ABZ absorption in a recently reported modeling analysis addressed to identify factors associated with changes in ABZ pharmacokinetics in humans [36].

On the other hand, different feeds may (a) induce changes in the pH and microbial population in the gut modifying the process of drug biotransformation, (b) decrease the stomach emptying rate (solid meals and fats), and (c) increase the gastric secretion of compounds associated with food digestion as well as the secretion of bile and other proteolytic enzymes into the duodenum and proximal small intestine. All of these effects may influence the rate and extent of absorption of lipid-soluble drugs [25]. It has been demonstrated that in growing pigs, the supplementation of corn oil reduced the gastric emptying time [37], which is inversely related to the energy content of the diet [38]. As the gastric emptying is the most important factor controlling the rate of access of the drugs to the site of absorption [39], a higher ABZ dissolution and delayed absorption as a consequence of delaying gastric emptying rate induced by the soya oil supplementation help to explain the observed results.

## 5. Conclusions

Under the experimental conditions tested here, fasting prior to ABZ treatment did not show any effect on the plasma pharmacokinetic behavior of ABZ metabolites in pigs compared to the animals fed ad libitum. Moreover, the addition of soybean oil to the pelleted food produced an enhanced and delayed absorption reflected in a higher systemic exposure between 12 and 48 h post-treatment. Although this effect may not be therapeutically relevant after ABZ administration as a single oral dose, the overall impact of the type and feeding conditions when ABZ is supplemented with the food for several days, should be cautiously evaluated. This may have particular relevance when dealing with the control of a resistant helminth parasite, where optimal drug exposure may be required to obtain a satisfactory therapeutic response.

## Figures and Tables

**Table 1:** Plasma pharmacokinetic variables (expressed as mean ± SD) for the albendazole sulfoxide (ABZSO) obtained after the oral administration of albendazole (ABZ) as a single dose (10 mg/kg, suspension) to pigs fed under different dietary regimens.

PK Parameters	Fasting	Pellet + Oil	Pellet
Cmax (µg/mL)	3.2 ± 0.8 ^a^	3.3 ± 1.5 ^a^	2.9 ± 0.6 ^a^
Tmax (h)	7.0 ± 2.1 ^a^	12.0 ± 6.0 ^a^	9.0 ± 2.1 ^a^
AUC_0–LOQ_ (µg.h/mL)	50.2 ± 11.1 ^a^	58.6 ± 14.2 ^a^	40.5 ±11.0 ^a^
AUC_12–48h_ (µg.h/mL)	24.3 ± 7.2 ^a^	37.7 ± 8.5 ^b^	17.5 ± 9.1 ^a^
AUC_0–∞_ (µg.h/mL)	50.7 ± 13.5 ^a^	59.7 ± 14.7 ^a^	40.6 ± 11.0 ^a^
MRT (h)	13.5 ± 3.4 ^a^	17.8 ± 4.0 ^a^	12.0 ± 1.9 ^a^
T½el (h)	6.4 ± 2.3 ^a^	6.7 ± 3.4 ^a^	5.3 ± 2.1 ^a^

Cmax, peak plasma concentration; Tmax, time to peak plasma concentration; AUC_0–LOQ_, area under the concentration versus time curve from time zero to the limit of quantification; AUC_12–48h_, area under the concentration versus time curve from time 12 to 48 h post-treatment; MRT, mean residence time; T½el, elimination half-life. ^a,b^ Different letters indicate statistically significant differences (*p* < 0.05).

**Table 2:** The pharmacokinetic parameters (expressed as mean ± SD) for albendazole sulphone (ABZSO_2_) after the oral administration of albendazole (ABZ) (10 mg/kg) to pigs fed under different dietary regimens.

PK Parameters	Fasting	Pellet + Oil	Pellet
Cmax (µg/mL)	0.8 ± 0.3	0.9 ± 0.2	0.8 ± 0.2
Tmax (h)	19.6 ± 6.7	26.0 ± 3.1	16.8 ± 6.5
AUC_0–LOQ_ (µg.h/mL)	15.5 ± 4.4	18.2 ± 4.1	13.1 ± 4.3
MRT (h)	18.5 ± 4.0	24.6 ± 4.9	16.9 ± 3.5
T½el (h)	4.2 ± 2.0	4.4 ± 2.3	4.5 ± 2.3

Cmax, peak plasma concentration; Tmax, time to peak plasma concentration; AUC0–LOQ, area under the concentration versus time curve from time zero to the limit of quantification; MRT, mean residence time; T½el, elimination half-life. No statistically differences were observed in the parameters among groups.
